# de novo transcriptomic profiling of differentially expressed genes in grass halophyte *Urochondra setulosa* under high salinity

**DOI:** 10.1038/s41598-021-85220-7

**Published:** 2021-03-10

**Authors:** Anita Mann, Naresh Kumar, Ashwani Kumar, Charu Lata, Arvind Kumar, Babu Lal Meena, Dwijesh Mishra, Monendra Grover, Sonam Gaba, C. Parameswaran, Nitin Mantri

**Affiliations:** 1 ICAR-Central Soil Salinity Research Institute, Karnal, Haryana India; 2 ICAR- Indian Agricultural Statistical Research Institute, New Delhi, India; 3 ICAR- National Rice Research Institute, Cuttack, Odisha India; 4 School of Science, RMIT University, Victoria, Australia

**Keywords:** Biotechnology, Plant sciences

## Abstract

Soil salinity is one of the major limiting factors for crop productivity across the world. Halophytes have recently been a source of attraction for exploring the survival and tolerance mechanisms at extreme saline conditions. *Urochondra setulosa* is one of the obligate grass halophyte that can survive in up to 1000 mM NaCl. The de novo transcriptome of *Urochondr*a leaves at different salt concentrations of 300–500 mM NaCl was generated on Illumina HiSeq. Approximately 352.78 million high quality reads with an average contig length of 1259 bp were assembled de novo. A total of 120,231 unigenes were identified. On an average, 65% unigenes were functionally annotated to known proteins. Approximately 35% unigenes were specific to *Urochondra*. Differential expression revealed significant enrichment (*P* < 0.05) of transcription factors, transporters and metabolites suggesting the transcriptional regulation of ion homeostasis and signalling at high salt concentrations in this grass. Also, about 143 unigenes were biologically related to salt stress responsive genes. Randomly selected genes of important pathways were validated for functional characterization. This study provides useful information to understand the gene regulation at extremely saline levels. The study offers the first comprehensive evaluation of *Urochondra setulosa* leaf transcriptome. Examining non-model organisms that can survive in harsh environment can provide novel insights into the stress coping mechanisms which can be useful to develop improved agricultural crops.

## Introduction

The food supply for increasing human population is rapidly declining with scarcity of land and fresh water for agriculture. This problem is further exacerbated by climate change that increases the incidence of various abiotic stress factors. Thousands of species of naturally stress-resistant plants have evolved with time, some of which have already been domesticated by humans and are considered minor crops. Broader cultivation of these minor crops will diversify plant agriculture and human diet and will, therefore, help in improving global food security. More research is, therefore, required for understanding and utilizing these naturally stress-resistant plants. Technologies are now available to rapidly improve plant genetics, with the goal of increasing productivity while retaining stress resistance and nutritional value^1^. Although a lot of information is available for salt tolerance mechanism of glycophytes such as *Arabidopsis thaliana*^2^ and halophytes such as *Thellungiella halophile,* yet the specific regulatory mechanisms that enable halophytes (naturally stress-resistant plants) to survive in extremely saline habitats has still not been completely elucidated.

Globally, approximately 20% of cultivated land is affected by soil salinization^3,4^, and it is predicted to reach 50% in 2050^5^. In India, salt-affected soils occupy an area of about 6.73 million ha of which saline and sodic soils constitute roughly 40% and 60%, respectively. Soil salinity is an increasing problem for agriculture, affecting the most productive crop areas of the world. The agricultural production is, therefore, threatened by unavailability of more agricultural land and few salt-resistant varieties. This makes it imperative to develop salt stress resistant crop plants.

One of the ways to make salt stress tolerant crop plants is to genetically engineer them to sustain their growth and productivity in challenging environments. This requires exploring gene pool of wild relatives of crop plants. This can be achieved by analysing global gene expression of salt tolerant species to reveal the underlying regulatory and metabolic mechanisms. Glenn et al. (1999)^6^ have suggested a number as high as 6000 species of halophytes and additionally, the eHALOPH^7^, Halophyte Database currently identifies more than 1500 species as salt tolerant, without labelling them as halophyte. The main halophytes explored for physiological and molecular studies for salt tolerance mechanism include *Sueda*^8,9^, *Spartina species*^10^, *Salicornia brachiate*^11^ and *Atriplex *etc*.* Still the salt tolerant grasses (STGs) that have not been explored for gene network analysis may be utilised to elucidate their mechanisms of survival at high salt concentrations. *Urochondra setulosa* (Poaceae) is a rhizomatous perennial grass that grows in dune slacks, banks of salt-water creeks and saline flats. It is distributed in Northern Africa through Arabia, southern coast of Pakistan (Sindh) and Northwest India^12^. It can survive up to 1000 mM NaCl but genomically, its mechanism of survival in high saline environment has not been explored. Next-generation sequencing coupled with bioinformatics tools provides a platform for high-throughput analysis of key metabolic pathways involved in stress response and adaptation. Here, we used RNA sequencing to identify the genes present in a highly salt tolerant non-model plant species, *Urochondra setulosa* and identify the genes associated with salt tolerance.

## Materials and methods

### Plant material

**Figure 1:**
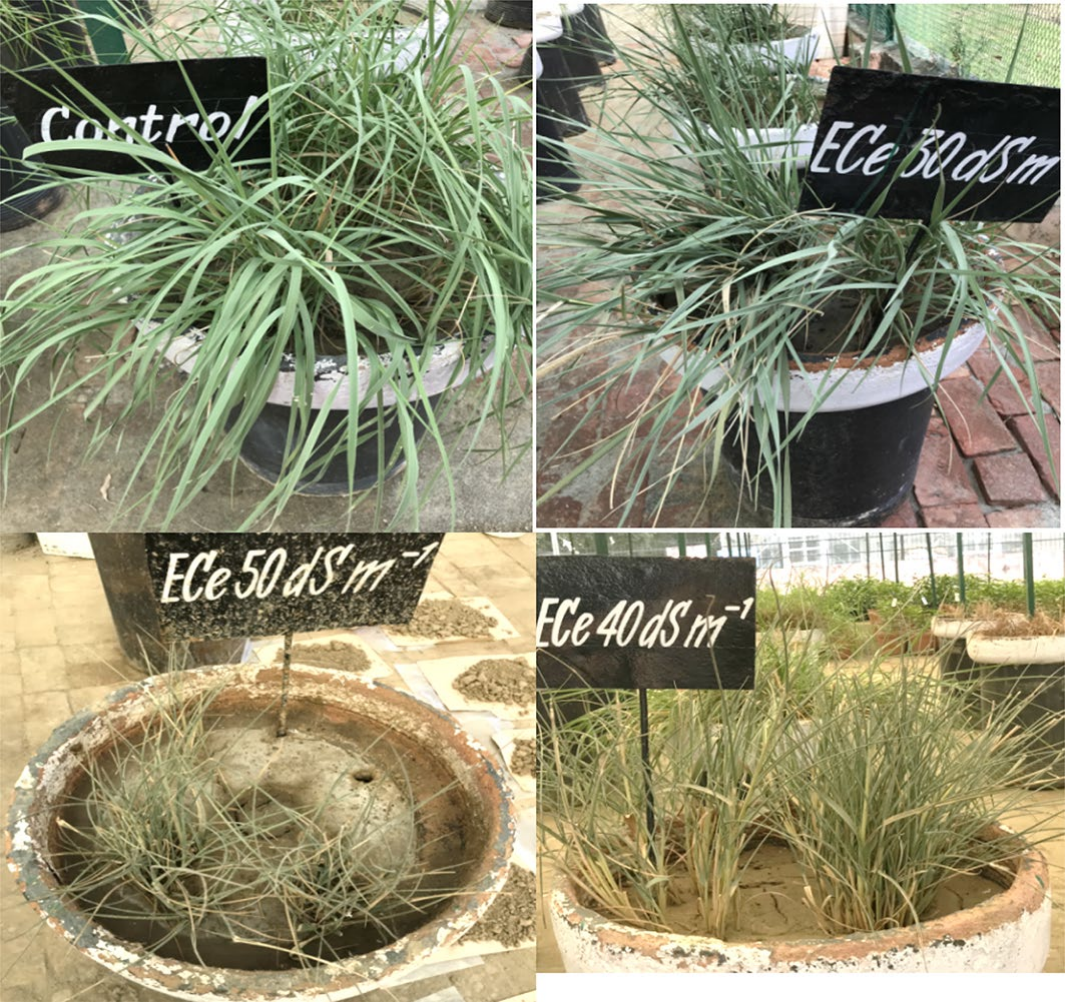
*Urochondra setulosa* plants at different treatment levels.

The halophyte grass *Urochondra setulosa* was collected from Rann of Kutch (the natural saline habitat of the halophyte) Bhuj, Gujarat, India and raised through root cuttings in pots and then established in lysimeters filled with sandy soil in a screen house under natural conditions at ICAR—Central Soil Salinity Research Institute (CSSRI), Karnal, Haryana, India (Fig. 1). The treatment levels of salinity i.e. ECe ~ 30 dS/m (~ 300 mM NaCl), ECe ~ 40 dS/m (~ 400 mM NaCl) and ECe ~ 50 dS/m (~ 500 mM NaCl) were maintained through saline water irrigation (3:1 chloride dominated salinity) in three replicates along with one set of control. Leaves were harvested at vegetative stage after 48 h of saline irrigation. In terms of soil salinity, ECe of 4dS/m equals to approximately 40 mM NaCl^13^. All methods were performed following the relevant guidelines and regulations.

### RNAseq library preparation

RNA sequencing libraries were prepared with Illumina-compatible NEBNext Ultra Directional RNA Library Prep Kit (NEB, USA) as per manufacturer’s instructions. mRNA was isolated from total RNA (100 ng to 1 µg) and subjected to first strand synthesis in the presence of Actinomycin D (Gibco, life technologies, CA, USA) followed by second strand synthesis. HighPrep magnetic beads (Magbio Genomics Inc, USA) were used to purify double-stranded cDNA and after end-repairing and adenylation, it was ligated to Illumina multiplex barcode adapters as per NEBNext Ultra Directional RNA Library Prep Kit protocol.

The adapter-ligated fragments were enriched through amplification of adapter-ligated cDNA using Indexing-PCR (37 °C for 15mins, denaturation at 98 °C for 30 s followed by 15 cycles of 98 °C for 10 s, 65 °C for 75 s and 65 °C for 5mins. The final PCR product (sequencing library) was purified with HighPrep beads, quality was checked on Qubit fluorometer (Thermo Fisher Scientific, MA, USA) and fragment size distribution was analysed on Agilent 2200 Tapestation.

### de novo transcriptome sequencing and data processing

Plant samples in two biological replicates per treatment were used for sequencing on Illumina HiSeq sequencer at Genotypic Technology, Bangalore (India) to generate 150 base pair length paired-end reads. On an average 46.08 million raw sequencing reads for each treatment samples were generated which were processed for quality assessment and low-quality filtering before the assembly. The raw data generated was checked for the quality using FastQC^14^ and pre-processing of the data was done with Cutadapt^15^. Data with Q-score above 30 (> 99.9% correct) was taken as high quality data.

Processed reads were assembled using graph-based approach by Trinity program. The characteristic properties, including N50 length, average length, maximum length, and minimum length of the assembled contigs were calculated (Table 1). Clustering of the assembled transcripts based on sequence similarity was performed using CD-HIT-EST^16^ with 95% similarity between the sequences, reducing the redundancy without exclusion of sequence diversity and was used for further annotation and differential expression analysis. Processed reads were aligned back to the final assembly using Bowtie^17^ with end to end parameters.

**Table 1:** Assembly Statistics of unigenes in *Urochondra setulosa.*

	Assembled transcripts (*Urochondra*)	Clustered transcripts (*Urochondra*)
Number of transcripts identified	165,794	120,231
Maximum contig length	15,263	15,263
Minimum contig length	300	300
Average contig length	1,275.0 ± 1,061.5	1259
Median contig length	853.5	1555
Total contigs length	211,394,399	155,760,927
Total number of non-ATGC characters	0	0
Contigs ≥ 200 bp	165,794	123,715
Contigs ≥ 500 bp	123,385	92,646
Contigs ≥ 1 Kbp	77,203	56,845
Contigs ≥ 10 Kbp	29	21
N50 value	1851	1819

### Differential expression analysis

DESeq, a R package was used for differential expression calculation^18^. DESeq provides methods to test for differential gene expression using a prototype based on negative binomial distribution and a shrinkage estimator for the distribution's variance. Sequencing (uneven library size/depth) bias among the samples was removed by library normalization using size factor calculation in DESeq.

### GO annotation and pathway analysis of DEGs

Uniprot and KEGG pathway databases were used for functional annotation of the transcripts for homology approach through BLAST against “*viridiplantae*” data. Transcripts were assigned with a homolog protein from other organisms, if the match was found at e-value less than e-5 and minimum similarity greater than 30%. KAAS server was explored for assigning pathway analysis. Since no reference genome is available for the halophyte, *Urochondra setulosa,* hence for pathway identification *Zea mays*, *Oryza sativa japonica*, *Musa acuminata* and *Dendrobium officinale* were considered as reference organisms being monocots from same family *Poaceae*.

### Validation of DEGs by qPCR

Quantitative real-time PCR (qPCR) analysis was performed on CFX96 real-time PCR (Bio-Rad) to validate the differentially expressed transcripts. Ten DEGs were randomly selected for qPCR analysis. Total RNA was isolated from the same sample as used for RNAseq and treated with the DNase I followed by cDNA synthesis using First Strand cDNA Synthesis Kit (Thermo Scientific). The primer sets for randomly selected DEGs were designed using an online Primer Quest tool from Integrated DNA Technologies (https://eu.idtdna.com/Primerquest/Home/Index) as listed in Table 2. Real-time PCR was performed with 20 µl PCR reaction mixture prepared according to the instruction provided with SsoAdvanced Universal SYBR Green Supermix (Bio-Rad). The qPCR of each gene was done in three technical replicates with three biological replicates. Actin was used as a reference gene and ΔΔCT method was used to calculate the fold change^19^.

**Table 2:** List of primers used for qPCR validation.

Sr no	Gene ID	Forward primer (5′–3′)	Reverse primer (5′–3′)
1	Urochondra_DN32950_c0_g1_i1	TGGTGGAGACTTCAGACAAACG	GCATGTGATCCCAGAGGTAAG
2	Urochondra_DN38352_c1_g1_i4	GGTCTAGTTCGAGTGTACTGTG	TTCTTGCATGGTCCACAGG
3	Urochondra_DN29996_c0_g1_i1	CAGCAATAGTCCAGTAAGCGAG	TGCTCCATGCTTCCTTTCGG
4	Urochondra_DN40311_c0_g1_i1	CGAAGAGTCCATGCTGTGATTG	CTCTCCTTCTTTCCCTCTGGTAC
5	Urochondra_DN28631_c1_g1_i1	GCTGTACCTGAAGCTCTTCTAC	GCGACAGGAAATCCACTATG
6	Urochondra_DN40034_c6_g2_i3	TTCATCTTCTGGACGCTCAC	GTGAATGAATCTTGCGGATGCC
7	Urochondra_DN42336_c2_g1_i1	ATCGGATTCCTAAGCTGACGGAG	GCTGTGACAGGTGGTTGATAC
8	Urochondra_DN37709_c6_g2_i6	GTTACGAGAAATCCTCCTGGTG	GCAGAAGTGTCACTTGAAGCC
9	Urochondra_DN35411_c4_g1_i4	GGTGAGTGGTGACAATGAGAG	CTCCGCAACGTAGTCATGTAAC
10	Urochondra_DN32108_c0_g1_i1	TGCCAGATAGGGACGATACTG	GTCGTCATCTTCCTCCTGAATC

## Results

### Read statistics and de novo assembly

**Figure 2:**
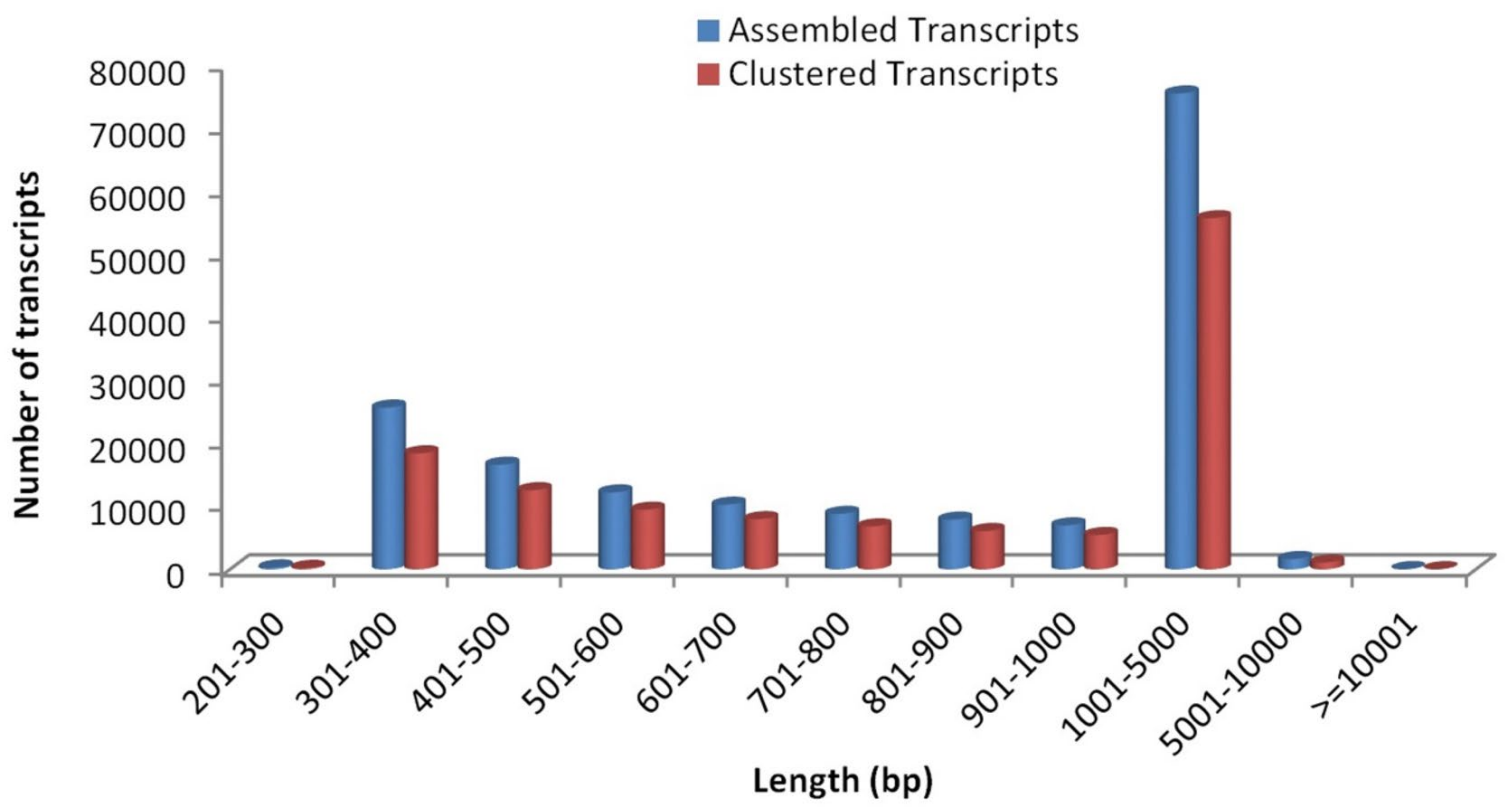
Transcripts length distribution for *Urochondra sequences.*

Approximately 368.70 million raw reads were generated by Illumina paired-end transcriptome sequencing and used for the downstream analysis. After the quality control, on an average 95.66% of clean reads (high quality reads having Phred score > q30) were obtained. The high-quality RNA-seq reads were de novo assembled into transcripts using Trinity, as no reference genome sequence is available for *Urochondra setulosa*. The transcriptome coverage efficiency was assessed, in absence of reference genome, by relating the unique genes with the closest available transcriptome in de novo sequencing^20,21^. The trinity assembly of high quality reads resulted in 165,794 transcripts, further clustering resulted in 120,231 transcripts (in all treatments) with an average contig length of 1259 bp and N50 of 1819 bp for *Urochondra* samples (Table 1). Since the shorter sequences may lack a characterized protein domain or may be too short to show sequence matches, resulting in false negative results, the contigs which were less than 300 bp in length were excluded (Fig. 2). As a whole, a total of 96.79 million, 91.76 million, 90.05 million and 90.09 million raw reads were obtained from the control and salt treated transcriptome libraries of *Urochondra*, respectively (Table 3). More than 94% high-quality reads (clean reads) were obtained for each group and used for downstream analyses.

**Table 3:** Statistics of transcriptome sequencing results in *Urochondra setulosa.*

Sample	Control	EC ~ 30 dS/m	EC 40 dS/m	EC 50 dS/m
Raw reads	48,399,217	45,882,055	45,026,242	45,046,274
Processed Reads	46,811,900	44,368,130	42,628,010	42,582,319
Alignment to clustered transcripts (%)	94.10	94.01	84.88	84.39
% of high quality data	96.72	96.69	94.67	94.54

### Similarity search and GO annotation

**Figure 3:**
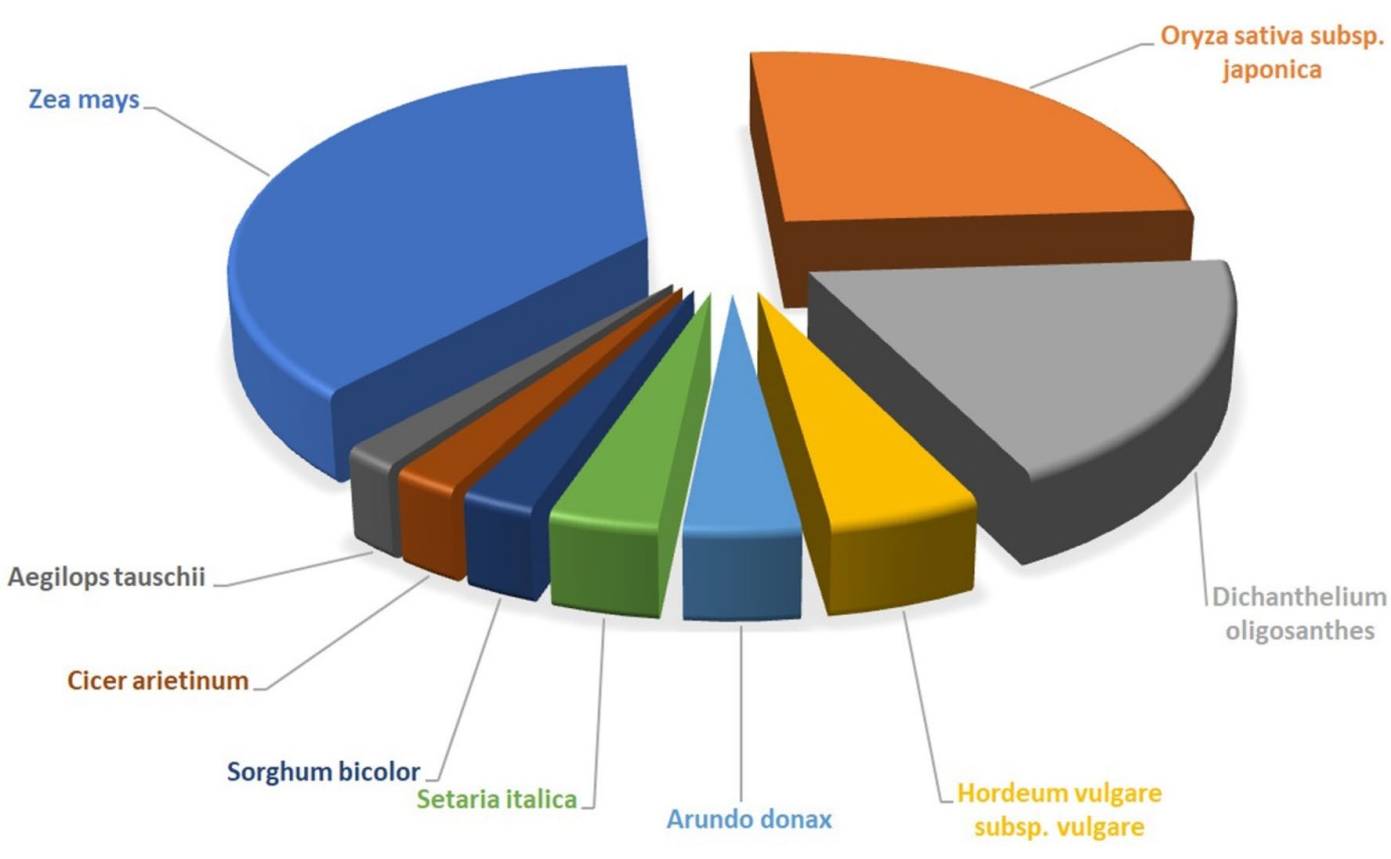
Sequence similarity index of *Urochondra* transcripts with other species.

The clustered high-quality reads were blasted against the *viridiplantae* database in NCBI. Of the 120,231 clustered transcripts, 78,775 transcripts (65.52%) were annotated against uniport database and remaining 41,456 (34.48%) transcripts could not be annotated due to lack of information for *Urochondra* in the database. The E-value distribution of the transcripts showed that 58.03% of the aligned transcripts had similarity with an E-value range of 1e−05 to 1e−60, whereas the remaining homologous sequences ranged from 1e − 5 to 0. As evident from the Fig. 3, most of the transcripts had a significant level of sequence similarity to *Zea mays *(32.31%), *Oryza sativa *(22.19%), *Dichanthelium oligosanthes *(16.28%), *Hordeum vulgare subsp. Vulgare *(4.53%), *Arundo donax *(3.46%),* Setaria italica *(3.26%), *Sorghum bicolor *(2.18%), *Cicer arietinum *(1.95%) and *Aegilops tauschii *(1.65%).

**Figure 4:**
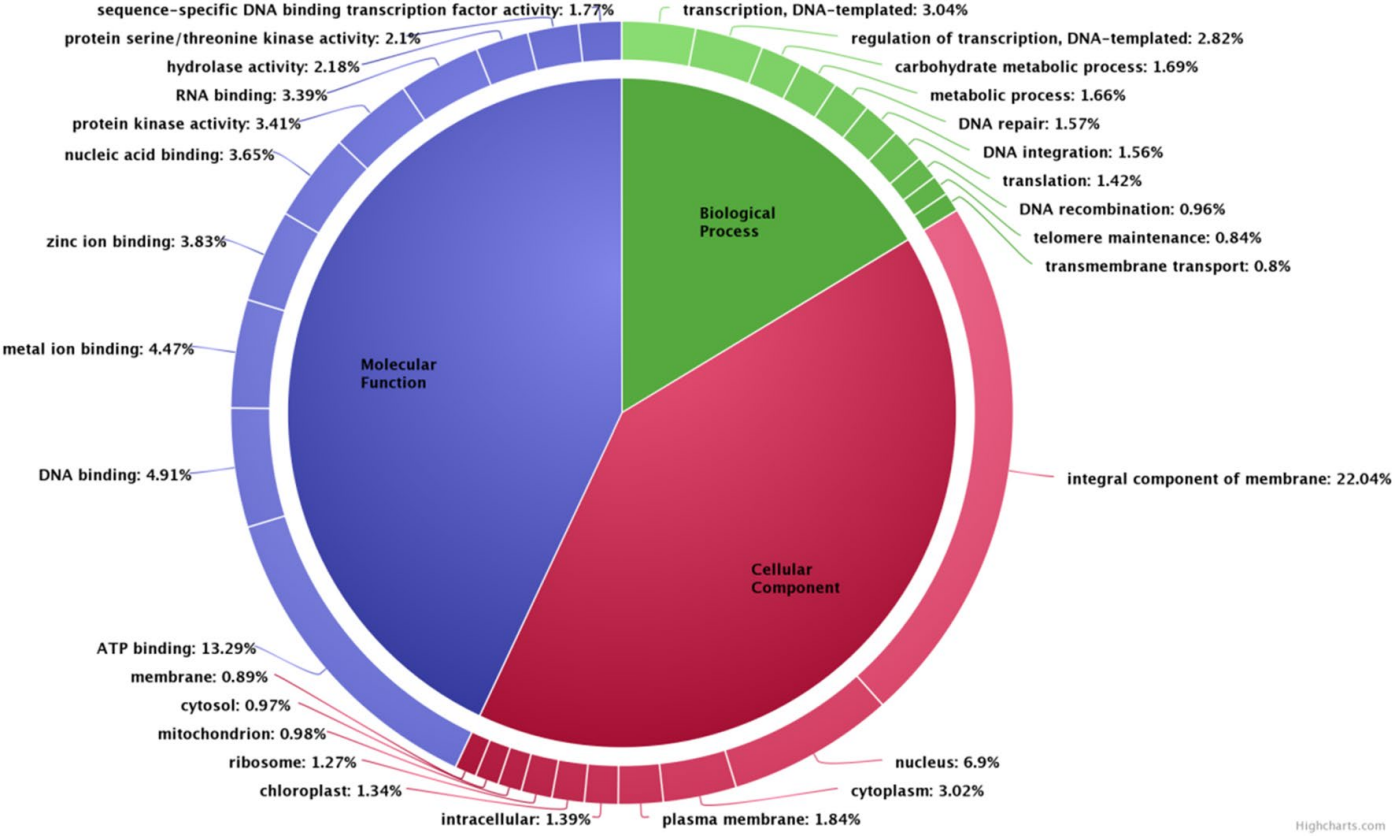
Frequency of top 10 abundant GO terms under biological process, molecular function and cellular component categories in *Urochondra setulosa.*

Based on the ontologies, the annotated transcripts were placed under three different categories of Biological processes (BP), Cellular component (CC) and Molecular function (MF) respectively. Functional enrichment of GO terms has been shown in Supplementary file S1.The most abundant category belonged to biological processes with 1931 terms with lowest 521 terms in cellular component and 1505 terms were obtained under molecular function. A donut chart representing the most abundant 10 terms from each of ontology has been given in Fig. 4.

**Figure 5:**
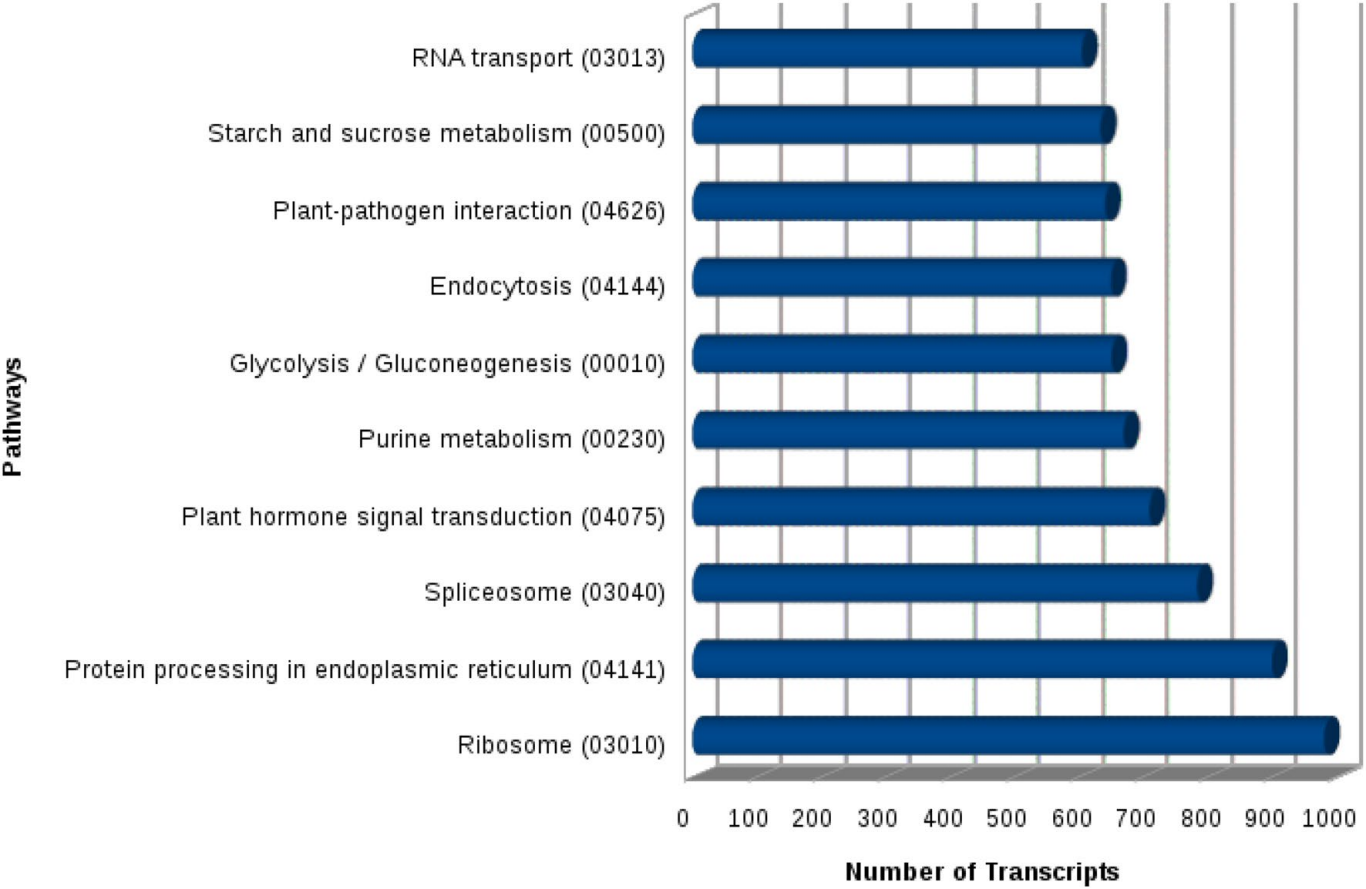
Top 10 most highly represented pathways in *Urochondra setulosa.*

KAAS server was used for functional annotation of these 18,953 unique transcripts and anthocyanin biosynthesis (1 transcript) was seen as the least in terms of the number of homologous transcripts (Fig. 5). The most abundant transcripts were observed for pathways of protein processing in the endoplasmic reticulum (900 transcripts) and ribosome (981 transcripts). KEGG enrichment for most common terms has been shown in Supplementary file S2.

### Identification of differentially expressed genes

**Figure 6:**
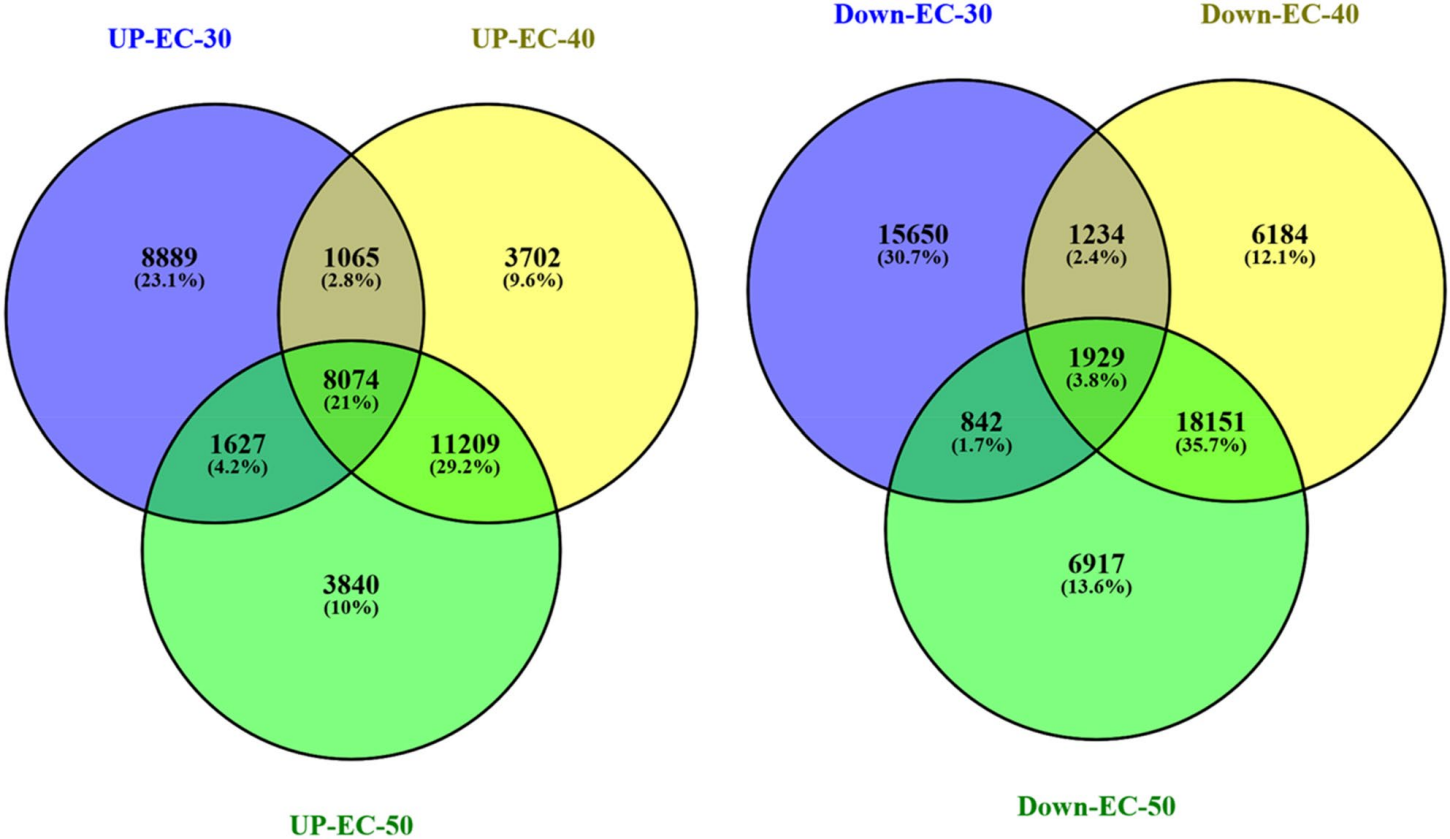
Venn diagram of DEGs at different saline levels in *Urochondra setulosa.* Constructed using Venny 2.1 (https://bioinfogp.cnb.csic.es/tools/venny/index.html).

**Figure 7:**
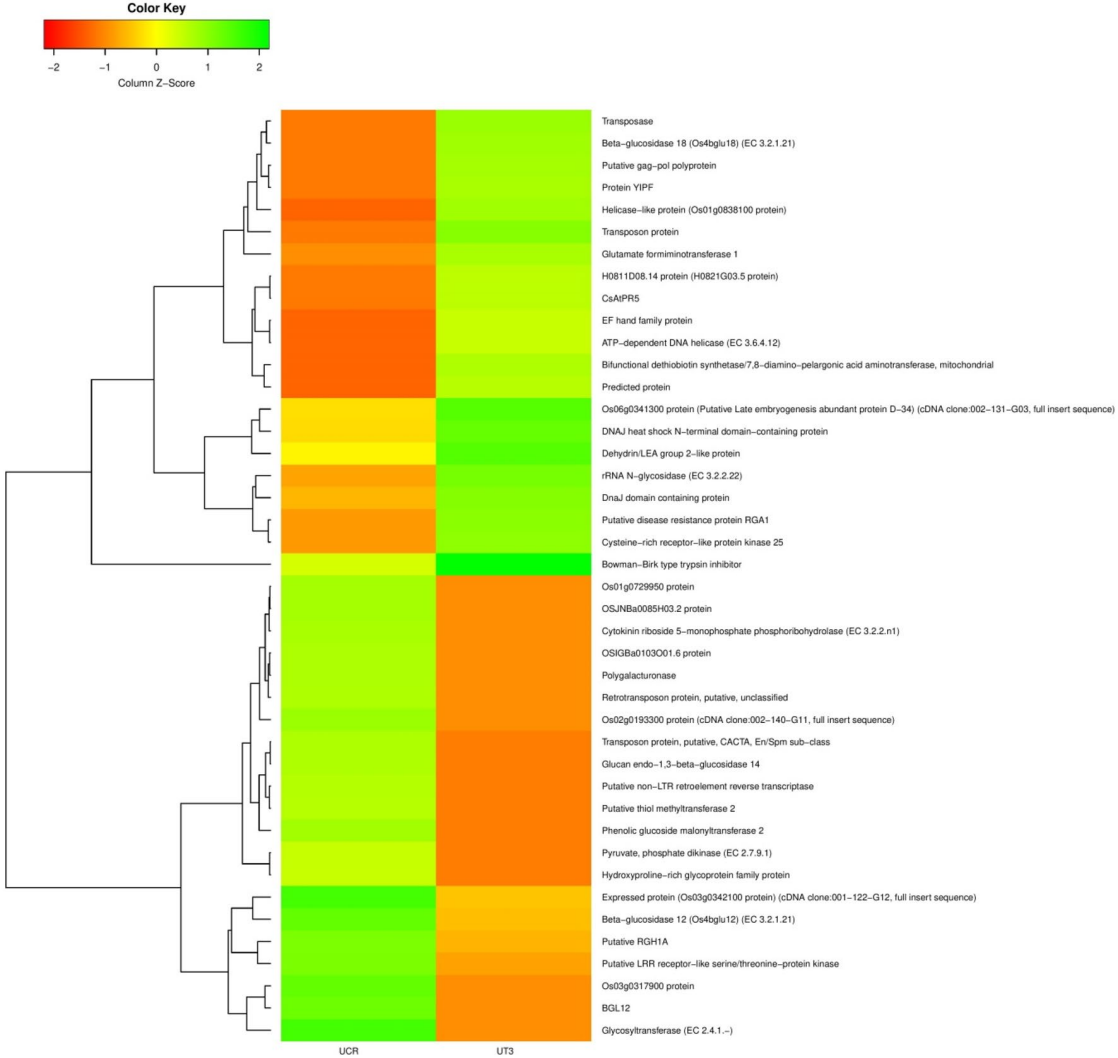
Heatmap representing top 20 up and down regulated transcripts generated using Clustvis (https://biit.cs.ut.ee/clustvis/).

DESeq normalized expression values were used to calculate fold change for a given transcript. The genes exhibiting a significant difference between treatment and control samples (at least two-fold changes with *P*-value ≤ 0.05) were considered to be differentially expressed. The regulation for each transcript was assigned based on log2fold change. The transcripts with a log2fold change less than − 1 were down-regulated, those with values greater than 1 were up-regulated and those between − 1 to 1 were termed as neutrally regulated. Total 345,729 transcripts were differentially expressed between control and treated samples of *Urochondra* with 120,231 trancsripts at EC 30 dS/m, 113,280 trancsripts at EC 40 dS/m and 112,218 at EC 50 dS/m w.r.t. control. Of the total transcripts expressed, 68,455 transcripts were up-regulated, 69,759 transcripts were down-regulated and 207,515 transcripts were neutrally regulated. The Venn diagram constructed using Venny 2.1 (https://bioinfogp.cnb.csic.es/tools/venny/index.html) reveals that 1,065 transcripts (2.8%) were commonly up-regulated at EC 30 and 40 dS/m, 11,209 (29.2%) transcripts between EC 40 and 50 dS/m and 1627 transcripts (4.2%) were common at EC 30 and 50 dS/m in *Urochondra* (Fig. 6). Similarly, 1234 transcripts (2.4%) were down-regulated at salinity levels of EC 30 and 40 dS/m, 18,151 transcripts (35.7%) at EC 40 and 50 dS/m and only 842 transcripts (1.7%) were commonly down-regulated at EC 30 and 50 dS/m. Heatmap was generated for top 20 up and down-regulated transcripts between control and treated *Urochondra* samples (Fig. 7) using Clustvis (http://biit.cs.ut.ee/clustvis/). In the heatmap, the green coloured bands identify high gene expression while the red colour represents the low gene expression level.

### Validation of DEGs

**Figure 8:**
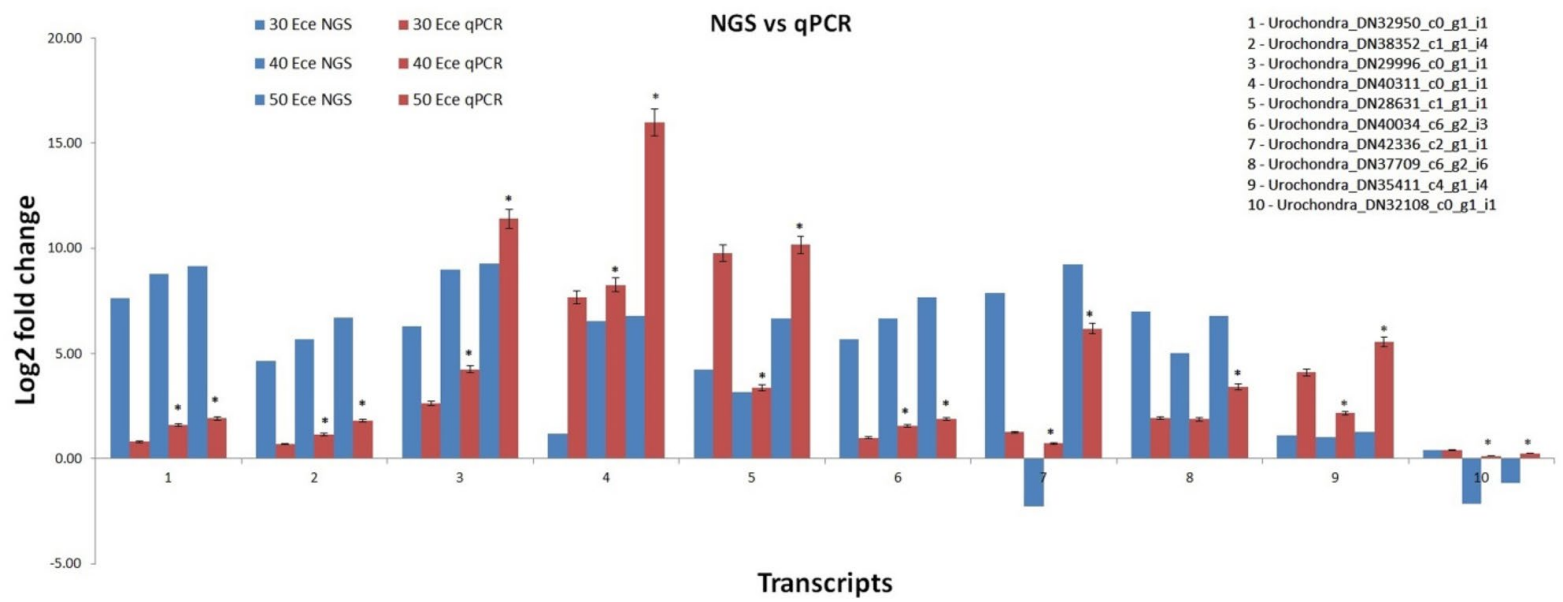
Comparative gene expression between qPCR analysis and RNA-Seq data (mean ± SE); * significantly different from the control (*P* < 0.05).

To confirm the reliability of RNA-Seq in identification of differentially expressed genes under salt stress, we performed the qPCR analysis of 10 randomly selected unigenes at salinity levels of ECe ~ 30 dS/m, ECe ~ 40 dS/m and ECe ~ 50 dS/m. A similar expression level was observed between qPCR results and RNA-Seq data but with some variations (Fig. 8). The results confirmed the reliability of RNAseq analysis. The qPCR expression analysis was performed to identify if pathways of the selected genes were involved in providing high-salinity tolerance to *Urochondra*.

### DEGs in response to high salinity

The genes expressed differentially in response to high salt concentrations in halophyte *U. setulosa* were found to be associated with various biological processes i.e. photosynthesis, MAP kinases, response to oxidative stress, osmotic stress, cellular response to oxidative stress, cellular response to stress, positive regulation of response to salt stress, stress-activated protein kinase signalling cascade, response to endoplasmic reticulum stress, trehalose metabolism in response to stress, priming of cellular response to stress, negative regulation of response to salt stress, response to cation stress and stress granule assembly.

### Photosynthesis

High salinity can severely affect the efficiency of photosynthesis due to decrease in photosynthetic pigments, decrease in unsaturation index, damaged photosynthetic system and ultimately reduction in the photosynthesis. Interestingly, in *Urochondra*, the expression of genes for the photosynthetic enzymes i.e*.* Ribulose bisphosphate carboxylase small chain (*Urochondra*_DN40184_c0_g1_i1), and ferredoxin (*Urochondra*_DN34936_c0_g1_i4), were found to be up-regulated in response to high salt stress (Supplementary file S3). High salinity also causes the degradation of photosynthetic pigments such as chlorophyll, and phycobilins. The expression of genes essential for the biosynthesis of chlorophyll pigment is down-regulated under the influence of salt stress. Remarkably, we observed that the expression of gene for the enzyme divinyl reductase (*Urochondra*_DN36240_c1_g1_i2) which converts 8-vinyl groups of different chlorophyll intermediates to ethyl groups was down-regulated with increasing salt stress. One more enzyme, Omega-6 fatty acid desaturase (*Urochondra*_DN40411_c2_g1_i3), which is involved in unsaturation of fatty acid of the chloroplastic membrane, was upregulated under salt stress.

### MAPK pathway

MAPK (Mitogen-activated protein kinases) are a particular class of serine/threonine protein kinases which are activated by various stresses such as salt, drought, cold etc. We observed the up-regulation of similar proteins i.e*.* protein phosphatase 2C (*Urochondra*_DN30190_c0_g2_i2), and Serine/threonine-protein kinase 10 (*Urochondra*_DN34126_c1_g1_i6) at high salinity i.e. ECe 30 dS/m, 40 dS/m and 50 dS/m (Supplementary file S4). Similarly, Copper-transporting ATPase RAN1 (*Urochondra*_DN38546_c0_g1_i1) which involved in MAP kinase pathway was also found to be up-regulated.

### Transcription factors

The total DEGs encoding transcription factors (TFs) increased with higher level of salinity i.e. 841, 1613 and 1623 at ECe 30 dS/m, 40 dS/m and 50 dS/m, respectively. These TFs mainly belong to various families such as BTB/POZ, WRKY, MYB, NAC, DREB, *AP2-EREBP*, *bHLH*, *bZIP*, *MADS*, and *SBP*. The DEGs encoding Myb-related protein Myb4 (*Urochondra*_DN37753_c2_g1_i9) and Myb family protein-like (DN35501_c2_g1_i2) were found to be up-regulated under ECe 30 dS/m, 40 dS/m and 50 dS/m salinity (Supplementary file S5). The TFs are also considered as important regulators under salt stress. The expression of transcription factor *WRKY40* (*Urochondra*_DN39291_c1_g1_i1) decreased with an increase in salinity level while the expression of ABA-responsive proteins (*Urochondra*_DN33328_c2_g1_i1) were found to be up-regulated with stress as reported in *Arabidopsis*^22^. It indicated that *WRKY40* may act as a negative regulator for ABA-responsive genes. In *Urochondra* too, the expression of *WRKY* was found up-regulated under salinity.

### Oxidative stress

**Figure 9:**
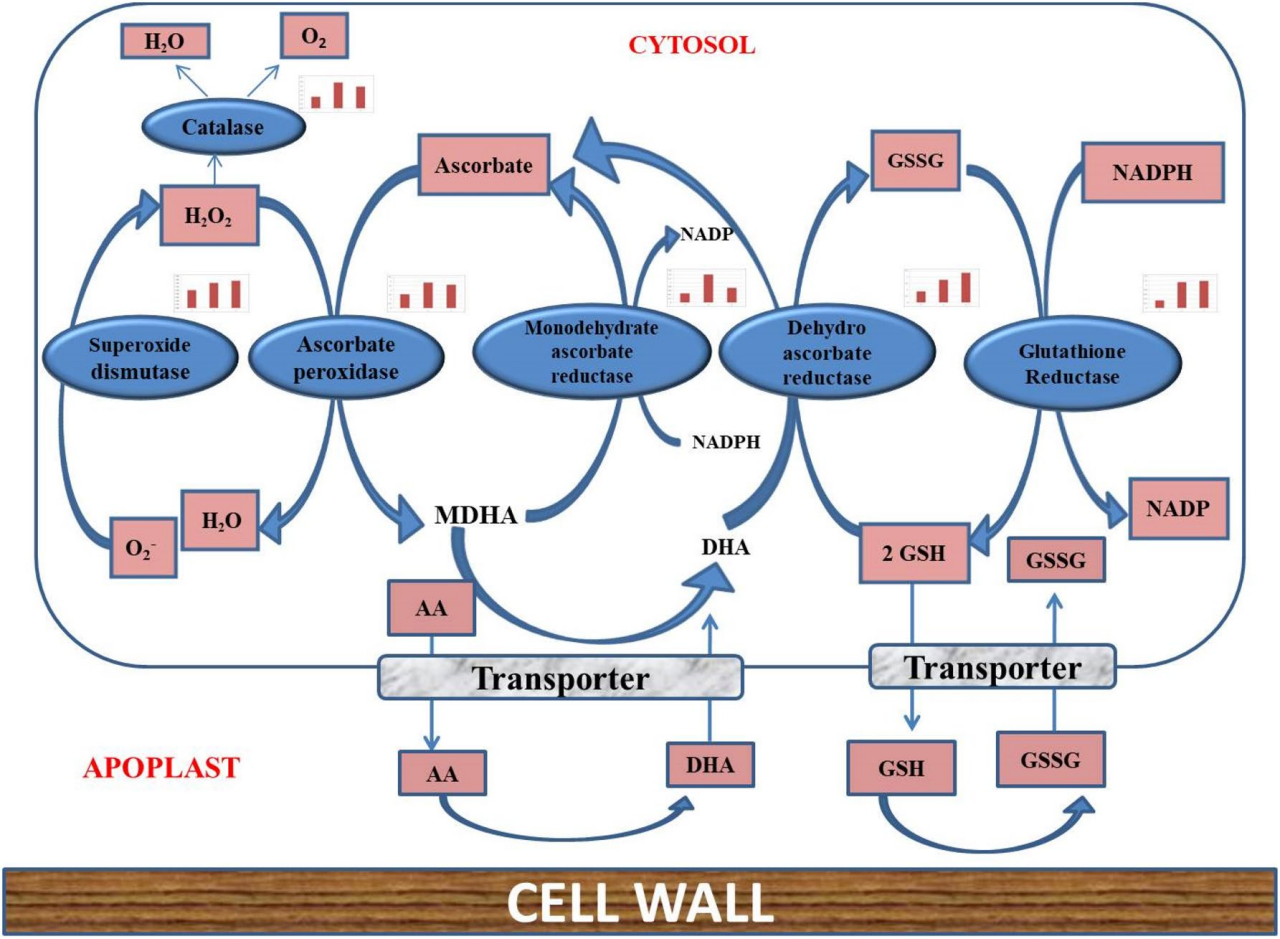
Differentially expressed genes of ROS pathway in response to saline stress.

DEGs in response to oxidative stress include peroxidases, catalases, Putative L-ascorbate peroxidase, Mitogen-activated protein kinase, Trehalose 6-phosphate phosphatase, Lactoylglutathione lyase, Methionine sulfoxide reductase, and alkaline alpha galactosidase. Oxidative stress is often associated with many stresses including salt stress. In present experiment, it was found that L-ascorbate peroxidase (*Urochondra*_DN34366_c0_g1_i1) and monodehydro ascorbate reductase (*Urochondra*_DN48599_c0_g1_i1) are up-regulated with increasing salt levels (Fig. 9) along with Catalase (*Urochondra*_DN29893_c0_g1_i1), and peroxidases (*Urochondra*_DN37483_c1_g1_i1).

### Compatible solutes

The transcripts *Δ-1-pyrroline-5-carboxylate synthase* (*Urochondra*_DN34937_c1_g1_i6), *Betaine aldehyde dehydrogenase* (*Urochondra*_DN24005_c0_g1_i1) and *Trehalose 6-phosphate phosphatase* (*Urochondra*_DN43873_c0_g1_i1) were found to be up regulated indicating accumulation of proline, glycine betaine and trehalose with increasing salt concentration.

### Transporter proteins

The transcriptomic profiling of *Urochondra* leaves revealed many DEGs encoding transporter proteins involved in various biological processes e.g., transmembrane transport, intracellular protein transport, metal ion transport, ATP synthesis coupled proton transport, vacuolar transport, sodium ion transport, amino acid transmembrane transport, potassium ion transport, and anion transmembrane transport (Supplementary file S6). Among them, DEGs encoding transmembrane transport were most abundant including calcium-binding mitochondrial carrier protein SCaMC-2-B, potassium exchanger-like protein, magnesium/proton exchanger 1, MRP-like ABC transporter, glutathione transporter, Na^+^/H^+^ antiporter (NHX). The expression of gene encoding Na^+^/H^+^ exchanger (*Urochondra*_DN33781_c0_g1_i1) and a larger number of ABC transporter genes was up-regulated along with the salinity stress in *Urochondra*.

### Cell wall modifying proteins

The cell wall components such as cellulose and hemicellulose are the major components which are responsible for the characteristic thickness of secondary cell wall. Differential gene expression analysis showed several cell wall related genes involved in the salt tolerance mechanism i.e. Cellulose synthase (*Urochondra*_DN40272_c2_g1_i1), Chitinase (*Urochondra*_DN40272_c2_g1_i1), Beta-galactosidase 15 (DN40296_c4_g2_i12), Pectin esterase (*Urochondra*_DN33727_c0_g1_i1), Xyloglucan endotransglucosylase/hydrolase (DN41141_c4_g3_i2), Polygalacturonase (*Urochondra*_DN29442_c0_g1_i1), cell wall peroxidase (*Urochondra_*DN34582_c4_g1_i1) (Supplementary File S7) and laccase (*Urochondra*_DN36255_c3_g1_i1). The upregulated cell membrane proteins are Chitinase, Beta-galactosidase, Pectinesterase, cell wall peroxidase and laccase while polygalacturonase and cellulose are down-regulated at higher levels of salinity.

## Discussion

In the present scenario of climate change, environmental stresses, particularly salinity stress limits growth, physiology and productivity of crop plants by the over-accumulation of toxic ions such as Na^+^ and Cl^−^. The effect of toxicity can be observed by measuring growth attributes, physiological/biochemical traits and yield characters. Most of the plants showed survival by excluding ions/salt from their cells or sequester them into the vacuoles and adopt various strategies to overcome the detrimental effects of stress. The plant responses along with the tolerance mechanisms of various crops have been in research from years but the information is available for few halophytes. Further, transcriptomics studies on salt related pathways/genes in halophytes e.g. *Sueda, Sporobolus, Atriplex, Spartina* etc. are being explored now for development of climate smart crops.

*Urochondra setulosa* is a native halophyte of extreme saline lands which is still unexplored for its salt tolerance mechanism. Few reports are available for its physiological and biochemical attributes at high salinity^23^ still the transcriptomic analysis at saline conditions is lacking. This study is the first report of transcriptomic studies of *Urochondra* at high salt levels for functional categorization of differentially expressed genes. Illumina paired-end transcriptome sequencing generated approximately 368.70 million raw reads with 95.66% of high quality reads having Phred score > q30. Compared with the other EST sequencing technologies, the RNA-Seq provides assembled and annotated high quality reads as studied in a number of plants such as *Suaeda salsa*^8,9^, *Spartina spp*^10,24^, pearl millet^25^, *Halogeton glomeratus*^26^*, Iris lacteal*^27^*,* sugar beet^28^ and *Withania Somnifera*^29^. Normalization of the library improves a number of annotated genes significantly and reduces the oversampling of ample transcripts^30^. DESeq normalized expression values were used to calculate fold change for a given transcript. The genes exhibiting a significant difference between treatment and control samples are considered to be differentially expressed.

While comparing the percentage of firstly reported annotated transcripts (65.52%) in *Urochondra* with previous studies in non-model plant species, it was found to be within the range; as reported 79% in *Suaeda salsa*^8^, 69.8% in American ginseng root^31^, 82% in Amaranthus^20^, 68% in *Spartina*^10^ and *Cicer*^32^. In *Suaeda salsa*, only 68.10% unigenes could be annotated where, 61.97%, 51.39%, 43.88%, 47.03%, 48.15%, 44.30% and 14.91% unigenes could hit genes in the RefSeq non-redundant proteins (NR), nucleotide (NT), Swissprot, KEGG, Eukaryotic Orthologous Groups (KOG), Pfam and GO database respectively^9^. In *Spartina maritime*, 6100 genes in leaves and 11,149 genes in roots were annotated against Poaceae database while in *Spartina alterniflora* and among these, 2938 genes were found in both root and leaf transcriptomes^10^. Similarly, in comparison to 35% unique transcripts obtained here, 8% unique transcripts were reported in maize^33^, 7% in Ginseng and Amaranthus^20,31^, 13% in *Spartina pectinata*^34^ and 35% in moth bean^35^.

More than 30% of DEGs of *S. alterniflora* has similarity of more than 90% to that of *Oryza sativa* followed by *Zea mays*, *Sorghum bicolor*, *Vitis viniflora* and *Arabidopsis thaliana*^24^. Similarly, in our studies, more than 32% of DEGs of *Urochondra* has similarity of more than 80% with *Zea mays* followed by *Oryza sativa*, *Dichanthelium oligosanthes*, *Hordeum vulgare* subsp. *Vulgare*, *Arundo donax*, *Setaria italica*, *Sorghum bicolor*, *Cicer arietinum* and *Aegilops tauschii*.

The qPCR expression analysis was performed to identify if pathways of the selected genes were involved in providing high-salinity tolerance to *Urochondra*. Previous studies reported correlation between the qPCR and DEG data^36,37^ and we also observed that all the validated genes had similar expression patterns that were consistent with the DEG data, but the fold change were not exactly the same.

Different processes of biological, cellular or molecular function having important role in various pathways were identified through functional annotation of differentially expressed genes. The mechanism of salt tolerance is a complex process having different physiological, cellular, metabolic and molecular responses. The role of genes in imparting salinity tolerance including osmolytes, protein kinases, transcription factors and ion transporters have been reported earlier^38,39^. Most important pathways identified for tolerance mechanism include SOS (salt overly sensitive) pathway, proline metabolism, calcium-signaling, plant hormone signalling and MAPK (mitogen-activated protein kinase) signalling^27^. Functional annotation of *S. alterniflora* revealed 26% of the ESTs belonging to stress-related proteins, followed by nucleic acid metabolism (17%) while a considerably high number (13%) of ESTs had no known protein function that included hypothetical and predicted proteins^40^. In *Suaeda salsa*, DEGs mapped to oxidative phosphorylation, ribosome, starch and sucrose metabolism, amino sugar and nucleotide sugar metabolism, protein processing in endoplasmic reticulatum and plant hormone signal transduction were significantly annotated^8^. Differential expression of unigenes in leaves (3,856 DEGs) and roots (7,157 DEGs) of *Beta vulgaris* depict different mechanism for salt tolerance at 200 and 400 mM NaCl^28^. GO and KEGG database enrichment analysis identified differentially expressed genes for signal transduction, protein phosphorylation and redox regulation in roots and leaves. Differential expression of 77,250 unigenes (48,682 upregulated and 28,568 downregulated) have been reported in *S. salsa* at 30% NaCl in leaves and roots, respectively^9^. Through KEGG pathway analysis, transcripts for amino acid metabolism, carbohydrate metabolism, fatty acid metabolism and nitrogenous compound were differentially expressed at 500 mM NaCl in *S. alterniflora*^24^. Similarly, in *Urochondra,* many differentially expressed genes for regulation mechanisms in the nucleus, such as DNA-templated transcription (regulation) and post transcriptional modifications were identified, suggesting salt stress activates gene regulatory networks.

High salinity can severely affect the efficiency of photosynthesis due to decrease in photosynthetic pigments; decrease in unsaturation index, damaged photosynthetic system and ultimately reduction in the photosynthesis. The photosynthetic enzymes such as ferredoxin and ribulose bisphosphate carboxylase are important for photosynthesis process but are down-regulated under salt stress^41^. The up-regulation of photosynthetic enzymes Ribulose bisphosphate carboxylase and ferredoxin in *Urochondra* might indicate the normal functioning of the photosystem under high salt concentrations. It is already established that the unsaturation index of higher plant decreases with an increase in the salinity level^42^. A number of genes are involved in the MAPK pathways which are responsible for the stress tolerance mechanism. One of the previously identified protein namely SOS2 (a ser/thr protein kinase) which interacts with a protein phosphatase 2C was found to be involved in the salt tolerance^43^. Copper-transporting ATPase RAN1 is essential for the biogenesis of ethylene receptor which is a negative regulator of ethylene biosynthesis^44^. Hence, up-regulation of Cu-transporting ATPase RAN1 results into the indirect inhibition of ethylene biosynthesis. In higher plants, the ethylene biosynthesis increases under stress leading to senescence^45^. However, here, the salt tolerance ability of this halophyte may be attributed to inhibition of ethylene biosynthesis.

In present experiment, the total DEGs identified that encode transcription factors (TFs) mainly belong to various families such as BTB/POZ, WRKY, MYB, NAC, DREB, AP2-EREBP, bHLH, bZIP, MADS, and SBP. Major plant transcription factor families such as bZIP, NAC, AP2/ERF, and MYB orchestrate regulatory networks underlying abiotic stress tolerance^46–48^. The DEGs that encode MYB transcription family were the largest group highly expressed under salinity. The Myb TFs are regarded as active players in the drought and salinity stress signalling in plants^49^. The DEGs encoding Myb-related protein Myb4 (DN37753_c2_g1_i9) and Myb family protein-like (DN35501_c2_g1_i2) were found to be up-regulated under 30 dS/m, 40 d/ m and 50 dS/m salinity (Supplementary file S5). The results obtained are consistent with the previous studies^25,27,50^. Genes encoding transcription factor MYB2 expressed differentially under drought stress in plant *Populus Canadensis*^50^*.* The TFs are also considered as important regulators under salt stress. The up-regulation of ABA-responsive proteins (DN33328_c2_g1_i1) in *Urochondra* along with down-regulation of transcription factor WRKY40 (DN39291_c1_g1_i1) with increasing salinity level indicated that WRKY40 may act as a negative regulator for ABA-responsive genes as have been reported in *Arabidopsis*^22^. In plant transcriptomics, WRKY are one of the major regulatory transcription factors having various roles in management of plant biotic and abiotic stress responses. It has been reported that WRKY transcription factors play an role in signalling networking of plants thereby acting as regulators as well as repressors^51^. WRKY40 binds directly to the W-box Cis-acting element and hence inhibits the gene expression of DREBIA, MYB2, AB14, AB15, ABF4, the ABA responsive genes. WRKY40, WRK70, WRK41, WRKY3, WRK15, WRK35, WRK18 were all down-regulated in response to drought in the drought sensitive chickpea genotype^36^. In contrast, WRKY24 and WRK23 were up-regulated in the tolerant genotype. In *Urochondra* too, the expression of WRKY was found up-regulated under salinity. WRKY TFs not only have the ability to modulate gene expression in response to plant stress but also in the plant defence mechanism as they form a highly interacting regulatory network^22^.

Role of ABA in management of plant stress responses includes reprogramming of cellular mechanism at transcriptional level including lipid and carbohydrate metabolism. Also the ABA dependent and independent pathways regulate the plant adaptation to adverse environments through AREB/ABF (ABA-responsive element binding protein/ABRE-binding factor) and DREB/CBF subfamily of the AP2/ERF transcription factors. It has been reported in *Arabidopsis* that overexpression of DREB/CBF regulates the signals for plant growth and development under different environments. Such transgenics although had enhanced tolerance to drought, salinity and low temperature but in addition, some developmental defects were also counterbalanced in this process^39,52^. The accumulation of proline and chlorophyll under salt stress is linked to zinc finger TF (ZFP3) in *Arabidopsis*^53^ due to integral role of transcription factors in linking salt sensory pathways with tolerance responses.

Plants have developed enzymatic (e.g., superoxide dismutase, peroxidase, and catalase) and non-enzymatic (e.g., ascorbate and glutathione cycles and some secondary metabolites) systems to eliminate ROS accumulation in the cells^54^. Biotic and abiotic stresses in plants cause overproduction of reactive oxygen species (ROS) in various cellular compartments causing oxidative stress^55^. As reported in halophyte, *S. alterniflora*, heat shock proteins of zeaxanthin epoxidase which is a precursor of ABA synthesis and involved in abiotic stress has been identified^10^. ROS production activates K^+^ and Ca^2+^ permeable channels of the plasma membrane which further catalyse the Ca^2+^ signalling events and results into program cell death^56^. Plant cells are capable of detoxification of ROS through its antioxidant machinery. One of the most prominent systems for removing hydrogen peroxide is an ascorbate–glutathione cycle which includes different antioxidative enzymes such as ascorbate peroxidase^57^. In present experiment, it was found that L-ascorbate peroxidase (DN34366_c0_g1_i1) and mono dehydro-ascorbate reductase (DN48599_c0_g1_i1) are up-regulated with increasing salt levels (Fig. 9). Catalase (DN29893_c0_g1_i1), and peroxidases (DN37483_c1_g1_i1) were also found to be up-regulated with an increase in the salt concentration as has been reported earlier in various studies^6,28,52^.

Another consequence of salt stress is an ionic imbalance which leads to ion-induced injury and disturbance in water homeostasis. In response to ionic imbalance, most of the halophytes and glycophytes accumulate a number of low molecular mass compatible solutes i.e. proline, betaine, and sugars to accommodate an excess of ions in the vacuole^58^. The significant enrichment of transcription factors for proton transport suggests the role of ion homeostasis and regulation in salinity adaptation of *Spartina alterniflora*^24^. In *Urochondra*, the up regulation of transcripts for proline, glycine betaine and trehalose indicates accumulation of these osmolytes with increasing salt concentration.

The transporter proteins expressed in grass halophyte *Urochondra* are in accordance with the previous studies^25,28,59^. De Vos et al.^59^ reported genes responding to salt stress in *Artemia* and identified the transporter proteins i.e. Lipid transporter proteins, metal transporters proteins, ion transporter proteins and salt-dependent proteins. Genes involved in transport and kinase activities are highly enriched under salt stress in both root and leaf trancriptome of *S. alterniflora*^24^*.* NHX proteins are ubiquitous membrane proteins which play important role in the ion homeostasis by sequestration of additional Na^+^ in vacuoles or removal of excess Na^+^ from the cells^60^. In our study, the expression of gene encoding Na^+^/H^+^ exchanger (*Urochondra*_DN33781_c0_g1_i1) was up-regulated along with the salinity stress. Similarly, in a previous study, the *Arabidopsis ABC transporter*, AtABCG36, was found to promote salinity stress adaptation by reducing the shoot sodium content^61^. *S. alterniflora* maintains the salt tolerance ability by regulating uptake and accumulation of Na^+^, K^+^ and Cl^−^ through up-regulation of selective stress-related transporters^24^. Interestingly, in our data, up-regulation of a larger number of ABC transporter genes was observed in *Urochondra* at moderate or higher levels of salinity.

It is believed that cell wall associated proteins i.e. expansins, xyloglucan endo-β-transglucosylases/hydrolases, endo-1,4-β-D-glucanase, play a key role in cell wall expansion and enlargement. Along with these extensibility proteins, some cell-modifying proteins are also important for the cell wall plasticity which involves polygalacturonase, pectin acetylesterase, pectin methyl esterase, cellulase etc.^62^. Cinnamoyl CoA reductase and cinnamyl alcohol dehydrogenase, genes for cellulose and cellulase synthase and glycosyl transferase were identified in *Spartina* transcriptome^10^. Remarkably, in present experiment, we observed the up-regulation of Chitinase (*Urochondra*_DN36592_c2_g1_i1), Beta-galactosidase (*Urochondra*_DN40296_c4_g2_i12), Pectinesterase (*Urochondra*_DN33727_c0_g1_i1) and down-regulation of polygalacturonase (*Urochondra*_DN29442_c0_g1_i1), cellulase (*Urochondra*_DN42285_c4_g3_i5) with the higher level of salinity. The cell wall components such as cellulose and hemicellulose are the major components which are responsible for the characteristic thickness of secondary cell wall. In the process of secondary cell wall formation, the precursor of lignin get cross-linked into the cell wall which depends on the presence/absence of cell wall peroxidases and laccases^63^. In our experiment, we found the up-regulation of cell wall peroxidase (*Urochondra*_DN34582_c4_g1_i1) and laccase (*Urochondra*_DN36255_c3_g1_i1) transcripts with increase in the salinity level.

Briefly, the response of *Urochondra setulosa* is slightly different from other studied halophytes by maintaining membrane stability and better plant growth at higher salinity. Differential gene expression analysis depicts that, RUBISCO activity is upregulated and hence photosynthesis is not inhibited by salinity. Other reports are also available in *U. setulosa* with enhanced gas exchange efficiency and plant biomass at salinity^23^. In other halophytes, e.g. in *Suaeda salsa*, down-regulation of photosynthesis was observed with upregulation of antioxidant genes at 30% salinity^9^. Ion homeostasis mechanism of *U. setulosa* is similar to that observed in other highly salt tolerant halophytes, *Thellungiella halophile* and *Spartina alterniflora* through upregulation of H^+^-ATPases, Na and K transporters under salinity^24,64^. *U. setulosa*, being a perennial halophyte find opportunity for further exploring salt tolerance pathways in a way that salt cress (*Thellungiella halophila*) is able to tolerate high salinity levels for shorter periods only and not able to survive at high salt concentrations for longer durations^64^.

## Conclusions

This is the first report of comprehensive transcriptome profiling of the grass halophyte *Urochondra setulosa* under increasing salt concentrations. In the Poaceae family, *Urochondra* belongs to subfamily Chloridoideae which is not explored genomically in comparison to other major grass species like rice, wheat, maize or sorghum. Therefore, in-depth comparative transcriptomic analysis of leaf tissues were performed at ECe 30 dS/m (~ 300 mM NaCl), 40 dS/m (~ 400 mM NaCl), and 50 dS/m (~ 500 mM NaCl). The de novo transcriptome sequences were analyzed using the GO and KEGG tools which allowed annotation of a considerable portion of the transcriptome. Analysis of species without any reference genomic databases, transcriptome is the finest critical tool to explore the genic compartment among plants. One of the aspects of this work was to build a reference transcriptome using NGS technology for further annotation and identification of specific genes providing survival at high salt concentration as well as genes of economic and evolutionary importance. The main emphasis was on how the regulation of transcription factors and signalling transcripts are influenced by salinity. Moreover, validation of RNAseq results was performed using real time PCR analysis.

The up-regulation of genes for photosynthetic enzymes, MAPK pathway, transcription factors, transporter proteins, antioxidative enzymes, cell membrane proteins and enzymes for synthesis of compatible solutes with increasing levels of salinity suggested the reasons for salt tolerance ability of halophyte *Urochondra*. This study will be instrumental in understanding the putative role of genes involved in salt stress response in other grasses. Future investigation using functional validation tools will aid to identify the individual role of these genes, their mechanisms, pathways involved and application in the field for developing salt tolerant genotypes. The de novo transcriptome generated in this study will provide a useful source of reference sequence. The information generated here will additionally contribute to the biology of halophytes and expand the corresponding knowledge within the plant kingdom. Additionally, this work identifies potential genes involved in salt tolerance in STGs which can be used as donors for other halophytes or grasses such as cereal crops.

## Supplementary Information

Supplementary Information 1.

Supplementary Information 2.

Supplementary Information 3.

Supplementary Information 4.

Supplementary Information 5.

Supplementary Information 6.

Supplementary Information 7.

## Data Availability

The datasets generated during and/or analyzed during the current study are available from the corresponding author on reasonable request. Raw sequencing data have been deposited at NCBI under SRA accession number PRJNA561259. The Transcriptome Shotgun Assembly project has been deposited at DDBJ/EMBL/GenBank under the accession GJAG00000000. The version described in this paper is the first version, GJAG01000000.
